# SeqPredNN: a neural network that generates protein sequences that fold into specified tertiary structures

**DOI:** 10.1186/s12859-023-05498-4

**Published:** 2023-10-03

**Authors:** F. Adriaan Lategan, Caroline Schreiber, Hugh G. Patterton

**Affiliations:** Center for Bioinformatics and Computational Biology, Stellenbosch University, Stellenbosch, 7600 South Africa

**Keywords:** Protein design, Proteins, Structural biology, Neural network, Deep learning, Machine learning, Inverse folding, Sequence prediction

## Abstract

**Background:**

The relationship between the sequence of a protein, its structure, and the resulting connection between its structure and function, is a foundational principle in biological science. Only recently has the computational prediction of protein structure based only on protein sequence been addressed effectively by AlphaFold, a neural network approach that can predict the majority of protein structures with X-ray crystallographic accuracy.

A question that is now of acute relevance is the “inverse protein folding problem”: predicting the sequence of a protein that folds into a specified structure. This will be of immense value in protein engineering and biotechnology, and will allow the design and expression of recombinant proteins that can, for instance, fold into specified structures as a scaffold for the attachment of recombinant antigens, or enzymes with modified or novel catalytic activities.

Here we describe the development of SeqPredNN, a feed-forward neural network trained with X-ray crystallographic structures from the RCSB Protein Data Bank to predict the identity of amino acids in a protein structure using only the relative positions, orientations, and backbone dihedral angles of nearby residues.

**Results:**

We predict the sequence of a protein expected to fold into a specified structure and assess the accuracy of the prediction using both AlphaFold and RoseTTAFold to computationally generate the fold of the derived sequence. We show that the sequences predicted by SeqPredNN fold into a structure with a median TM-score of 0.638 when compared to the crystal structure according to AlphaFold predictions, yet these sequences are unique and only 28.4% identical to the sequence of the crystallized protein.

**Conclusions:**

We propose that SeqPredNN will be a valuable tool to generate proteins of defined structure for the design of novel biomaterials, pharmaceuticals, catalysts, and reporter systems. The low sequence identity of its predictions compared to the native sequence could prove useful for developing proteins with modified physical properties, such as water solubility and thermal stability. The speed and ease of use of SeqPredNN offers a significant advantage over physics-based protein design methods.

## Background

Levinthal and colleagues pointed out that, although the combination of all dihedral and allowed rotation angles in a protein was astronomical, the protein folded into a stable structure in solution within microseconds [1]. Anfinsen’s ribonuclease refolding experiments further underscored that this structure was solely dependent on the amino acid sequence of the protein [2].

The rate of generation of genomic data far outstrips the rate of solving protein X-ray crystallographic structures. This disparity has resulted in a bottleneck in the full functional interpretation of genomic data [3]. The development of algorithms to accurately predict protein structure from amino acid sequence was promoted by the bi-annual Critical Assessment of Techniques for Protein Structure Prediction (CASP) challenge [4]. This has resulted in spectacular advances in the success of ab initio predictions, and AlphaFold [5] and RoseTTAFold [6] now approach crystal structure accuracy. Many other methods are now available to accurately predict protein structures, including DMPFold [7], a deep learning model capable of predicting structures of proteins from underrepresented protein families, I-TASSER-MTD [8], which assembles template protein fragments into protein domains for multi-domain proteins, and PAThreader [9], another deep learning model that identifies remotely homologous protein templates that are used to significantly improve the structures predicted by AlphaFold. The reverse of the problem, namely the prediction of an amino acid sequence that will fold into a specified protein structure, is an evolving field, and holds significant promise for the advancement of human health, polymer science and biotechnology, by the design of bespoke enzymes, structural proteins and interacting sub-units. To date the inverse folding problem has been addressed largely using physics-based methods to identify amino acid sequences that minimize the conformational energy of a backbone scaffold, with the aim of finding a sequence for which the target structure is the most stable conformation. This requires the calculation of pairwise energies between millions of amino acid side chain rotamers, resulting in a very high computational cost for protein design [10]. However, the success rate for producing stable proteins using these protocols may be as low as 6% [11] and many researchers screen hundreds to thousands of designs to find a few functional proteins [12].

Machine learning can be applied to design proteins much more efficiently, by exploiting the structural data of almost 200 000 proteins deposited in the Protein Data Bank [13]. Natural proteins have sequences that are near-optimal for stabilizing their folded state [14]. A neural network that successfully applies the principles of protein folding encoded in natural proteins could potentially generate protein sequences with a higher success rate using significantly fewer computations. Additionally, natural selection has optimized natural proteins for solubility and function, which would allow machine learning methods to produce proteins that are less likely to aggregate and are more flexible than the most-stable sequence.

A few tools addressing the inverse folding problem using neural networks have recently appeared, including ProDCoNN [15], a convolutional neural network model, and ProteinSolver [16], a graph neural network model. Here we present SeqPredNN, which, unlike ProDCoNN and ProteinSolver, is based on a simple deep multilayer perceptron trained with sequence features distilled from sequences with less than 90% identity in the Protein Data Bank (PDB). We present a novel method for representing the structural context of amino acid residues using only the backbone atoms of 16 proximal residues. We show that this minimal representation of a small volume around each residue is sufficiently informative to predict sequences that fold into the target structure. Compared to other tools SeqPredNN generates amino acid sequences with lower identity to the original sequence. This property will allow greater versatility in enzyme design and in material science.

## Implementation

SeqPredNN was developed in Python version 3.9 and executed on Windows (Version 10 build 19,042.1348) and Linux (Ubuntu 20.4.2 LTR) platforms. The neural network is implemented using the Pytorch API. SeqPredNN comprises 3 programs: *Featurise* (for generating input features for the neural network from the PDB files), *Train_model* (for training new neural network models) and *Predict* (for predicting sequences from structural features and evaluating trained models). The programs, including the parameters of the pre-trained model are freely available at https://github.com/falategan/SeqPredNN. Before a user can predict amino acid sequences for protein structures, *Featurise* must be used to generate input features from PDB files containing the target structures. The user may then run *Predict* with the pretrained SeqPredNN model parameters to generate amino acid sequences for the target structures using the features generated with *Featurise*. The user may also use the features to train a new neural network model by running *Train_model*. All three programs are executed using a command line interface. Detailed usage instructions are included in the README file on the GitHub repository.

### Data processing

**Fig. 1:**
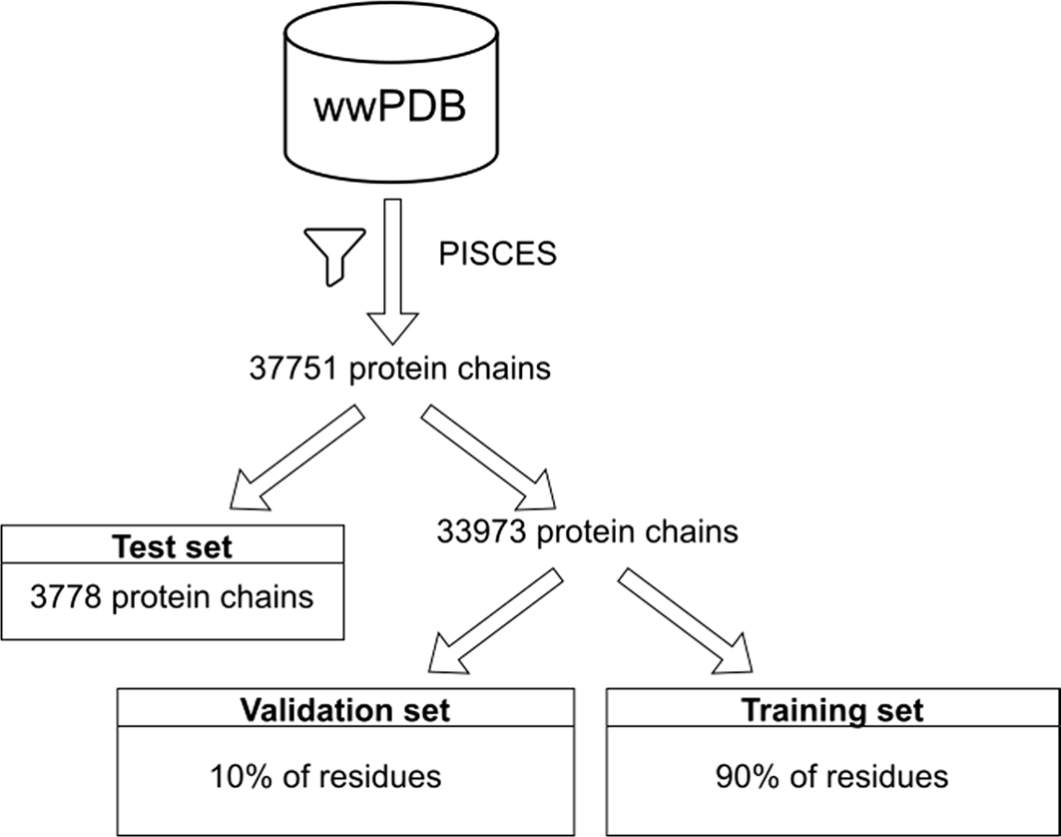
Flow diagram illustrating the sampling of protein structures to train, validate and test the SeqPredNN model

The data sampling procedure is summarised in Fig. 1. The protein structures from the entire PDB were filtered using the PISCES server [17] to obtain a nonredundant set of 33,973 protein chains with X-ray crystallographic structures at a resolution of less than 2.5 Å and a length greater than 40 residues, and where each protein chain has less than 90% similarity to any other chain in the dataset. Ten percent of the protein chains were randomly assigned to an independent test set. The rest of the chains were used to train the model. Ten percent of the residues in the protein chains of the training set were randomly assigned to a validation set that were only used to assess the model during training.

**Fig. 2:**
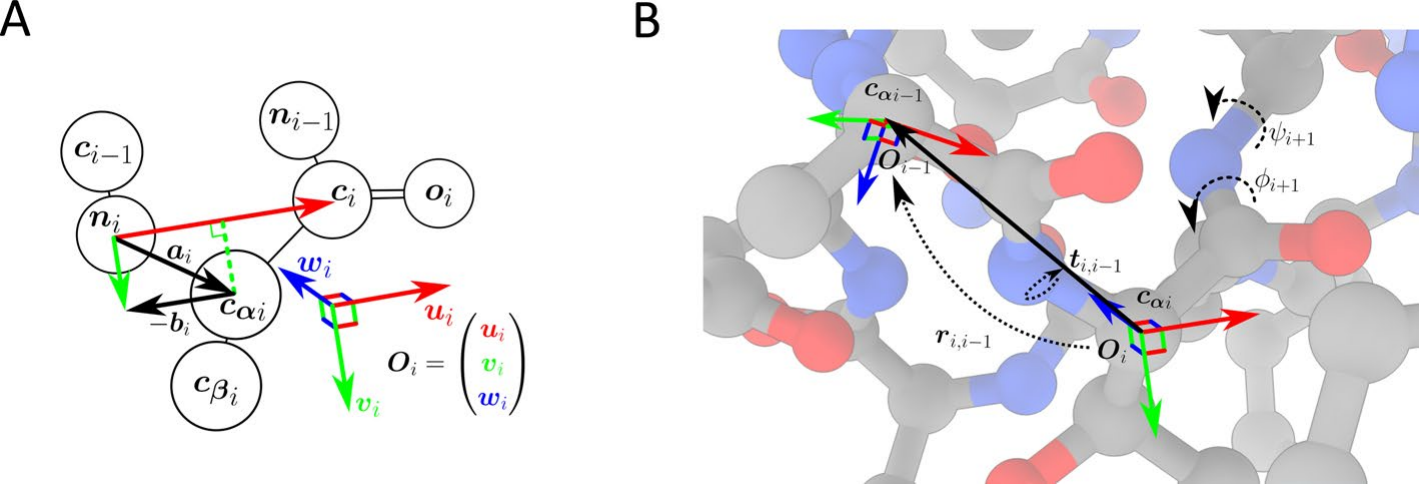
Derivation of structural features for an amino acid residue.** a** The orientation matrix for residue $i$ consists of 3 orthogonal unit vectors: ${{\varvec{u}}}_{i}$, ${{\varvec{v}}}_{i}$, and ${{\varvec{w}}}_{i}$. The first basis vector of the orientation matrix is the normalised vector from the backbone amide nitrogen to the backbone carbonyl carbon of residue $i$ in the PDB crystal structure, i.e., ${{\varvec{u}}}_{i}=\Vert {{\varvec{c}}}_{i}-{{\varvec{n}}}_{i}\Vert$. If ${{\varvec{a}}}_{i}$ is the vector from the amide nitrogen to the α-carbon ${{\varvec{c}}}_{\boldsymbol{\alpha }i}$, ${{\varvec{a}}}_{i}={{\varvec{n}}}_{i}-{{\varvec{c}}}_{\boldsymbol{\alpha }i}$, then the second basis vector ${{\varvec{v}}}_{i}$ is the normalised component of ${{\varvec{a}}}_{i}$ that is orthogonal to ${{\varvec{u}}}_{i}$. This vector is derived as the difference between ${{\varvec{a}}}_{i}$ and ${{\varvec{b}}}_{i}$, the projection of ${{\varvec{a}}}_{i}$ onto ${{\varvec{u}}}_{i}$ such that ${{\varvec{b}}}_{i}=({{\varvec{a}}}_{i}\bullet {{\varvec{u}}}_{i}) {{\varvec{u}}}_{i}$ and ${{\varvec{v}}}_{i}=\Vert {{\varvec{a}}}_{i}-{{\varvec{b}}}_{i}\Vert$**.** The third basis vector ${{\varvec{w}}}_{i}={{\varvec{u}}}_{i}\times {{\varvec{v}}}_{i}$. **b** The local structural environment of residue $i$ is represented by its relation to nearby residues $j$. The translation vectors ${{\varvec{t}}}_{i,j}$ are the vectors to the α-carbons of residues $j$, in the reference frame ${{\varvec{O}}}_{i}$ with basis vectors ${{\varvec{u}}}_{i}$, ${{\varvec{v}}}_{i}$ and ${{\varvec{w}}}_{i}$ with the origin on ${{\varvec{c}}}_{\boldsymbol{\alpha }i}$**.** Thus ${{\varvec{t}}}_{i,j}={{\varvec{O}}}_{i}\bullet \left({{\varvec{c}}}_{\boldsymbol{\alpha }j}-{{\varvec{c}}}_{\boldsymbol{\alpha }i}\right)$**.** The relative orientations of proximal residues are represented by rotation quaternions ${{\varvec{r}}}_{i,j}$, such that ${{\varvec{r}}}_{i,j}^{-1}{{\varvec{O}}}_{i}{{\varvec{r}}}_{i,j}= {{\varvec{O}}}_{j}$. The $\phi$ and $\psi$ dihedral angles of residues $i$ and $j$ are encoded as $\mathrm{sin}\phi$, $\mathrm{cos}\phi$, $\mathrm{sin}\psi$ and $\mathrm{sin}\psi$ values

The *Featurise* program derives local structural features for each amino acid residue in the dataset. These features represent the conformation of the protein backbone around a residue as the relative positions, orientations, and dihedral angles of nearby residues. The positions and orientations of these residues are expressed in a local coordinate system derived from the backbone geometry of each residue to ensure that the coordinates are invariant to translation and rotation of the entire protein, reducing the degrees of freedom in the representation of the protein structure. Figure 2a shows the derivation of the local coordinate system. The local backbone conformation around residue $i$ is represented as a set of translation vectors ${{\varvec{t}}}_{{\varvec{i}},{\varvec{j}}}$ from the α-carbon of residue $i$ to the α-carbon of each nearby residue $j$. Each translation vector consists of three coordinates in the local reference frame ${{\varvec{O}}}_{i,j}$**.** The orientation of each nearby residue is represented as a unit quaternion ${{\varvec{r}}}_{i,j}=a+b\widehat{{\varvec{i}}}+c\widehat{{\varvec{j}}}+d\widehat{{\varvec{k}}}$, which describes the rotation from the orientation ${{\varvec{O}}}_{i}$ to ${{\varvec{O}}}_{j}$. (Fig. 2b).

### Neural network model

**Fig. 3:**
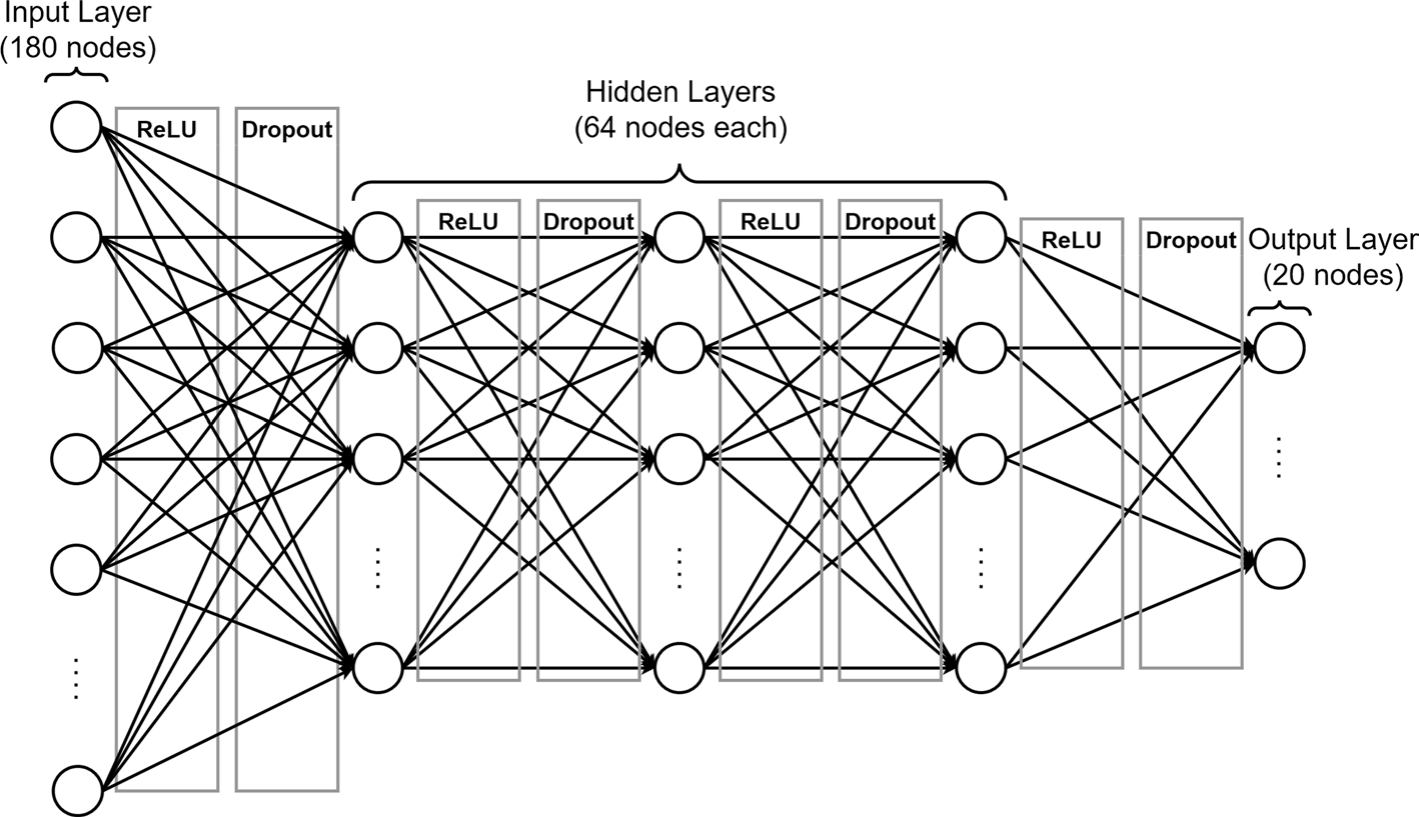
Schematic of the neural network architecture implemented in SeqPredNN

We trained the fully connected feed-forward neural network to classify a residue as one of the twenty standard amino acids based on the local structural features. The neural network consists of three hidden layers, with a rectified linear unit (ReLU) activation function and dropout [18] regularisation (with *p* = 0.5) between each layer (Fig. 3). The neural network outputs a SoftMax normalised prediction value for each of the twenty standard amino acids. The error of the model is quantified by the cross-entropy loss of the output, which is backpropagated to minimise the model by gradient descent. The Adam optimisation algorithm [19] optimises the model parameters with an adaptive learning rate. After extensive cross-validation on the validation dataset with multilayer perceptrons with two to five hidden layers, that are between 8 and 1024 nodes wide, we selected a neural network architecture with three hidden layers that are 64 nodes wide to optimise the sequence recovery rate, while also minimizing the number of learned parameters. The *Train_model* program allows users to train new neural network models, producing a parameter file containing the model weights and biases. We trained SeqPredNN using the training dataset described above, in batches of 4096 residues until the validation loss and sequence recovery converged after 200 epochs.

### Sequence prediction

The *Predict* program uses the model parameters generated by *Train_model* and the structural features generated by *Featurise* for each residue in a protein to predict an amino acid sequence that will fold into the specified conformation. It also evaluates newly trained models. We used *Predict* to determine the sequence recovery, precision and recall for each amino acid class.

### Validation of inverse folding

The native sequences as well as their matched SeqPredNN generated sequences for 662 protein chains, randomly sampled from the test set, were folded using the ColabFold interface to AlphaFold2 [20]. The default parameter of generating five structures per sequence was used, and the highest ranked structure was selected for further analysis.

We aligned the AlphaFold predicted structures for both the SeqPredNN generated and native sequences to the original PDB structure using TMscore [21]. The root-mean-square deviation (RMSD) and template modelling (TM) scores were then determined for each structure comparison. These measures were compared across the different independently folding protein domain classified by the CATH database [22] for each of the 427 single-domain proteins in the test set.

### Comparison with other tools

To compare SeqPredNN to two related programs, ProdCoNN and ProteinSolver, we predicted the sequence of seven proteins from different structure families using all three programs. These proteins were selected to represent a wide range of protein folds, including a trans-membrane barrel structure, a globular enzyme, and an all-alpha leucine zipper. We used the protein structures for histone H5 (PDB ID: 1HST), lipase EstA (PDB ID: 1I6W), rhodopsin (PDB ID: 1U19), general control transcription factor GCN4 (PDB ID: 1YSA), TATA-box-binding protein (PDB ID: 1YTB), Lysozyme C (PDB ID: 2CDS) and bromodomain-containing protein 2 (PDB ID: BRD2). We generated ProDCoNN predictions with the BBO_ID90 model, and ProteinSolver predictions using the ProteinSolver web server. ProteinSolver generates many sequences for a given structure. We obtained only the single highest confidence sequence for each structure by setting the number of sequences to one and the temperature factor to the minimum value (0.0001). We compare the sequence recovery of the three programs by determining the sequence identity between the generated sequences and the real sequences of the crystallised proteins according to a Needleman-Wunch pairwise alignment.

**Fig. 4:**
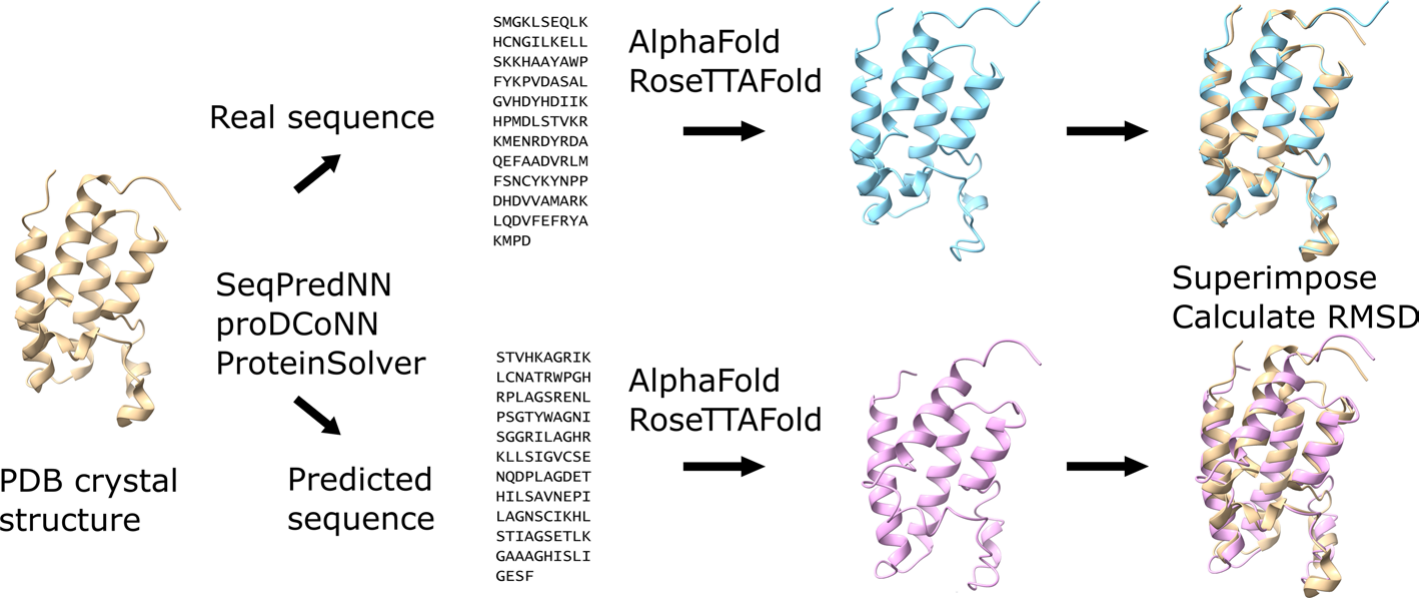
Generation of sequences that can fold into defined tertiary structures. A crystal structure was used to generate a sequence using SeqPedNN, proDCoNN or ProteinSolver, and both the generated and the real sequence were used to predict a tertiary structure using AlphaFold and RoseTTAFold. The RMSD of the structures predicted from the generated and real sequences was calculated to determine the ability of the generated sequence to fold into a structure identical to that of the real sequence

We predicted the folded structures of the generated sequences using AlphaFold and RoseTTAFold to estimate how close the folded conformation of each redesigned sequence is to the original crystallographic structure. AlphaFold and RoseTTAFold predictions were obtained using AlphaFold v2.1.0 Google Colab notebook and the Robetta server, respectively. We aligned the predicted structures of both the generated and native sequences to the original PDB structure using ChimeraX [23] and determined the root-mean-square deviation for the backbone α-carbons (C_α_–C_α_ RMSD) (Fig. 4).

## Results and discussion

**Fig. 5:**
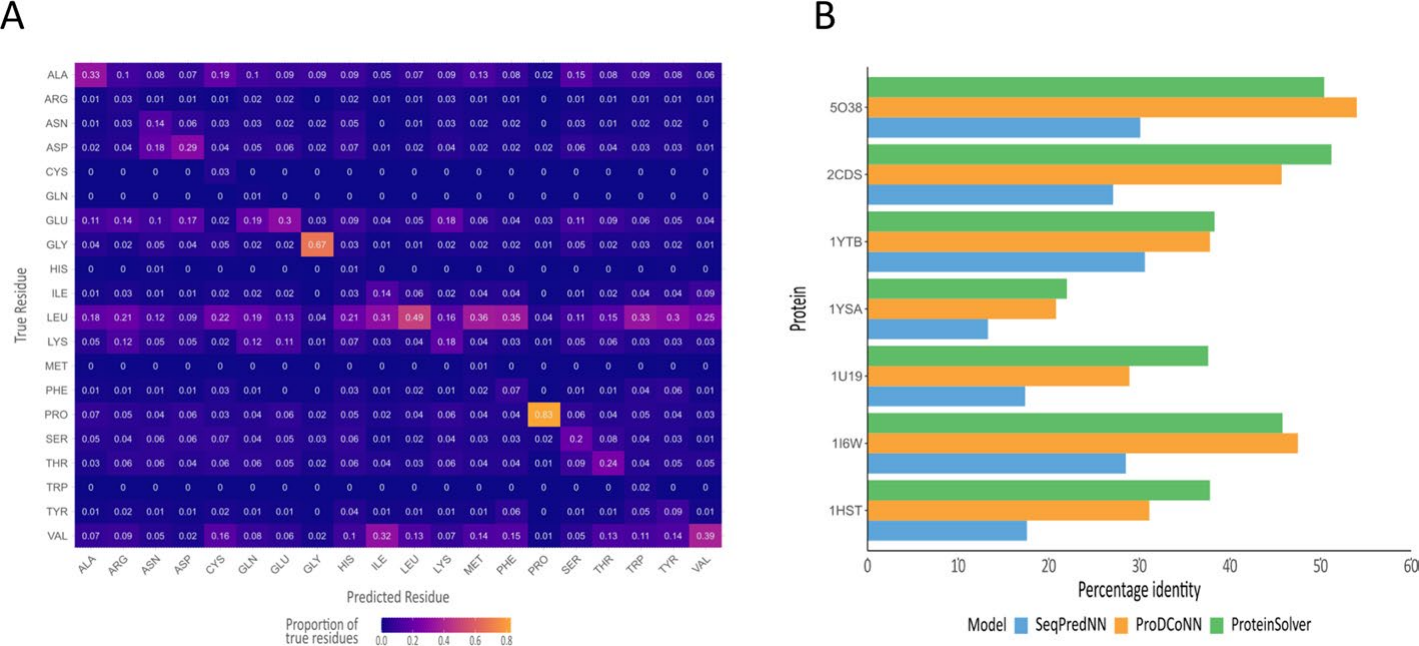
Accuracy of amino acid predictions. **a** Confusion matrix showing the empirical probability of the model predicting each amino acid (Predicted residue) given the original amino acid in the PDB structure (True residue). The values along the diagonal correspond with the recall for each amino acid class.** b** Percentage sequence identity of the sequences predicted by each model when aligned to the native sequence of the crystallographic structure

We evaluated the performance of SeqPredNN on the independent test set. The recall for each amino acid class shows that the model correctly identifies 67.5% of the glycine residues and 82.5% of proline residues in the test set (Fig. 5a). This is consistent with the conformational freedom of glycine and the constrained torsional angles of proline that have unique effects on the secondary structure of proteins. For the other amino acid classes, the model is more likely to suggest alternative amino acids than the residue in the original PDB structure. Overall, SeqPredNN achieves a sequence recovery of 28.4%. Sequence recovery is, however, a poor measure of model performance, because many protein pairs have highly similar structures even though they have very low sequence identity [24]. In fact, Kuhlman and Baker [14] have shown that the lowest energy sequence for a given backbone conformation has about 27% identity to the native structure. By comparison ProDCoNN and ProteinSolver are reported to have a sequence recovery of 46.5% and 32.0%, respectively. This is confirmed by the sequence identity between the true sequences and predicted sequences of the seven structures we use to compare the three tools (Fig. 5). The average sequence identity of the SeqPredNN predictions is 23.5%, 38.0% for ProDCoNN and 40.4% for ProteinSolver. Although the sequence identity of a predicted sequence is lower in the case of SeqPredNN compared to ProDCoNN and ProteinSolver, we regard this as a positive attribute. SeqPredNN’s ability to find sequences with low identity to the native protein is likely to be valuable for sampling sequences not found in biological systems. These unique sequences could confer new dynamic and physicochemical characters that may be useful in material science and biotechnological applications. ProteinSolver predictions with a higher “temperature” parameter shares this advantage. Sequence recovery reveals how well a model can identify residues in a crystal structure, but it is clearly insufficient for assessing inverse folding. For this the final folded structure of the predicted sequence needs to be known.

TM-scores for the fit of each AlphaFold prediction (native sequence and SeqPredNN generated sequence) to the original PDB were computed. For ease of comparison between the RMSD and the TM-Score, we present the difference between two structures as 1-TM which we will refer to as the residual-TM. This value is bounded on $$[{0,1})$$, where zero represents perfect agreement, and 1 represents infinite dissimilarity between structures.

**Fig. 6:**
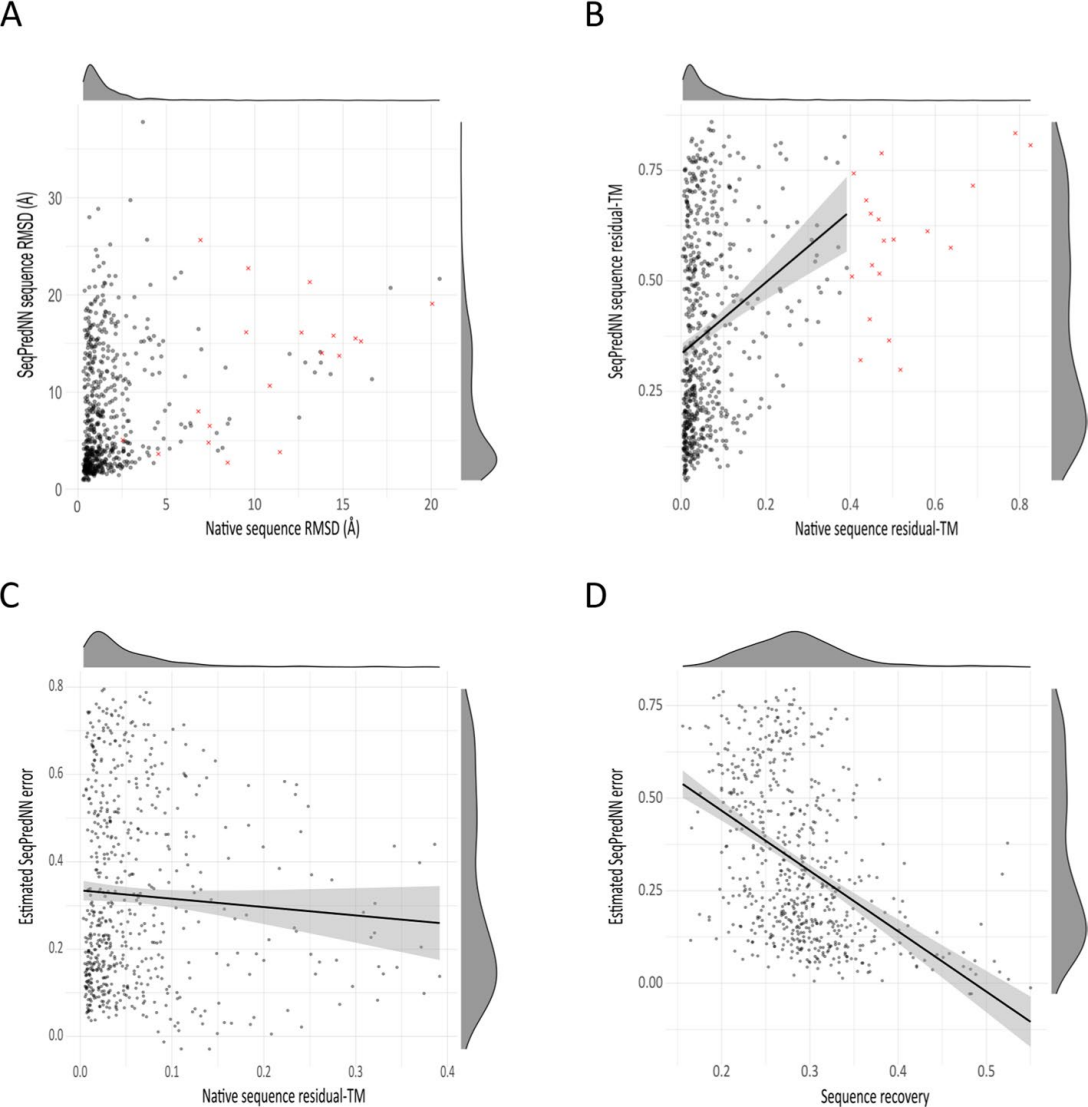
Divergence between AlphaFold models of SeqPredNN predicted sequences and the native crystal structure. Sequences with high residual-TM values for the AlphaFold model of the native sequence are indicated by red crosses. **a** Scatter plot of the RMSD for the AlphaFold models of 662 SeqPredNN compared to the RMSD of the AlphaFold models of the native sequence. **b** Scatter plot of the residual-TM for the AlphaFold models of 662 SeqPredNN compared to the residual-TM of the AlphaFold models of the native sequence. The regression line ${\text{residual-TM}}_{\text{SeqPredNN/AF}} =0.809\cdot {\text{residual-TM}}_{\text{Native}/\text{AF}}+0.335$ is shown with the standard error in the shaded area. **c** Scatter plot of the estimated divergence of proteins generated by SeqPredNN from the native structure (${\text{residual-TM}}_{{{\text{SeqPredNN}}/{\text{AF}}}}{-}{\text{residual-TM}}_{{{\text{Native}}/{\text{AF}}}} )$. The linear model $-0.191\cdot {\text{residual-TM}}_{\text{Native}/\text{AF}}+0.335$ is shaded with the standard error. **d** Scatter plot of the estimated SeqPredNN error against the proportion of residues predicted correctly in the sequencs. A least-squares line of best fit is shown shaded with the standard error

There is generally very little difference between the AlphaFold structure of a natural sequence and its x-ray crystallographic structure Fig. 6a. The Cα- Cα-RMSD for these alignments (Fig. 6a) have a median RMSD of 1.09 Å. Similarly, the median residual-TM (Fig. 6b) is only 0.038, confirming that AlphaFold produces highly accurate structure predictions for most protein sequences.

However, a small number of AlphaFold models do not agree with their crystal structures: 9 proteins have residual-TM values > 0.4. Protein pairs with a TM-Score of 0.6 (residual-TM = 0.4) have a more than 20% chance of belonging to entirely different CATH topologies, with the probability rapidly increasing to 63% as the residual-TM increases to 0.5 [25]. These proteins were excluded from the figures and from further statistics, since AlphaFold with a large error on the native sequence is unlikely to predict reliable structures for the SeqPredNN sequences of these proteins. Excluding these outliers resulted in a median residual-TM of 0.035.

The difference between the AlphaFold structures of SeqPredNN sequences and the crystal structure of the corresponding native sequence shows a much wider distribution than the native-sequence AlphaFold structures, with a median residual-TM of 0.334 and a median RMSD of 5.28 Å. Furthermore, Pearson’s $r$= 0.250 (95% CI = [0.174, 0.323], showing a significant correlation between the native residual-TM and the SeqPredNN residual-TM.

This relationship implies that there is a component of the SeqPredNN residual-TM that can be explained by the AlphaFold error on the native sequence. A linear regression was used to estimate the remainder of the error, i.e., the SeqPredNN error. This is the expected difference between the folded structure of a SeqPredNN sequence and the native crystallographic structure from which the SeqPredNN sequence was derived.

The resulting least-squares model estimates the residual-TM between the SeqPredNN AlphaFold model and the native crystal structure as ${\text{residual-TM}}_{{{\text{SeqPredNN}}/{\text{AF}}}} = 0.809 \cdot {\text{residual-TM}}_{{{\text{Native}}/{\text{AF}}}} + 0.335$. The estimated deviation of SeqPredNN structures from the original structure is the y-intercept, where the AlphaFold error is zero. This best estimate of the SeqPredNN error is a residual-TM of 0.33464 (95% CI = [0.312, 1.059]).

The estimate of the SeqPredNN error rests on the assumption that the AlphaFold error on predicted sequences is similar to the AlphaFold error on natural sequences. The difference between the residual-TM of each SeqPredNN sequence and the residual-TM of the native sequence provides an estimated SeqPredNN error for each sequence (Fig. 6c). This estimate successfully removes the influence of the AlphaFold error and shows no significant correlation with the AlphaFold error (Pearson’s $r$= 0.250, 95% CI = [− 0.140, 0.019]).

Furthermore, the estimated SeqPredNN error has a weak negative correlation to the sequence recovery (Fig. 6d). Thus, a SeqPredNN sequence that is highly similar to the native sequence is expected to fold into a conformation somewhat closer to the original conformation than a sequence with a low recovery.

**Fig. 7:**
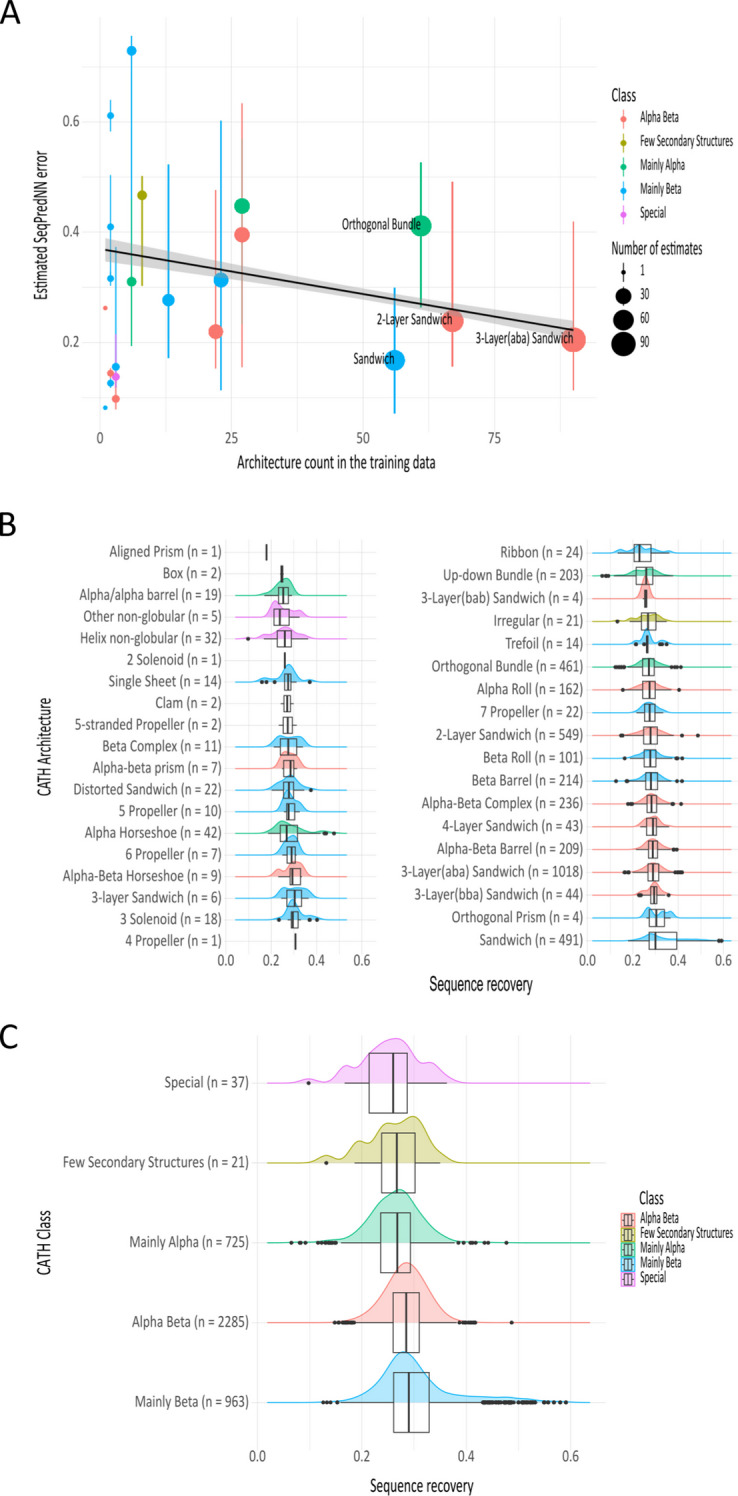
SeqPredNN performance across CATH domains.** a** Plot of the median estimated SeqPredNN error for CATH architectures with different frequencies in the training data. The medians are indicated by dots, and the area of the dots represent the number of single-domain proteins for each architecture in the test dataset. The vertical lines indicate the first and third quartile for each architecture. The least-squares regression line is fitted to all the individual protein domains datapoints (not presented here) with the standard error in the shaded region. **b** The distribution of sequence recovery rates for each CATH architecture represented in the test dataset as box-and-whisker plots were superimposed on the density of sequence recovery values. Outliers are presented as black dots. **c** The distribution of sequence recovery rates for each CATH class

The estimated SeqPredNN error also reveals the effect that the composition of the training data has on the model. A comparison between the protein domains at the Architecture level in the CATH hierarchy indicates that architectures that occur more frequently in the training data have lower estimated SeqPredNN errors (Fig. 7a). As a result, SeqPredNN tends to produce better predictions for common architectures like the 3-Layer(aba) Sandwich compared to underrepresented architectures like the Beta Complex.

Yet the distribution of the training data does not predict the performance of SeqPredNN across CATH architectures exactly. Figure 7b relates the distribution of sequence recovery rates across architectures for all single-domain proteins in the test set that are represented in the CATH database and demonstrates a slight difference between them. A Tukey–Kramer test on the 10 architectures with more than 100 sequences, confirms that there is a small, but highly significant difference in the means of different architectures. The mean sequence recovery of these groups differs by up to 8.42% recovery in the case of the Sandwich and Up-down Bundle architectures (95% CI 7.09%, 9.75%) This difference is not explained fully by the training data distribution and may be due to varying levels of structural disorder across the architectures.

Similarly, only a slight difference in the mean sequence recovery can be seen at the Class level in the CATH hierarchy (Fig. 7c). A Tukey–Kramer test shows significant differences in the mean sequence recovery of Mainly Alpha, Mainly Beta and Alpha Beta domains. The mean recovery of Mainly Beta is 4.17% points higher than the mean recovery of Mainly Beta domains (95% CI 3.57%, 4.77%).

**Fig. 8:**
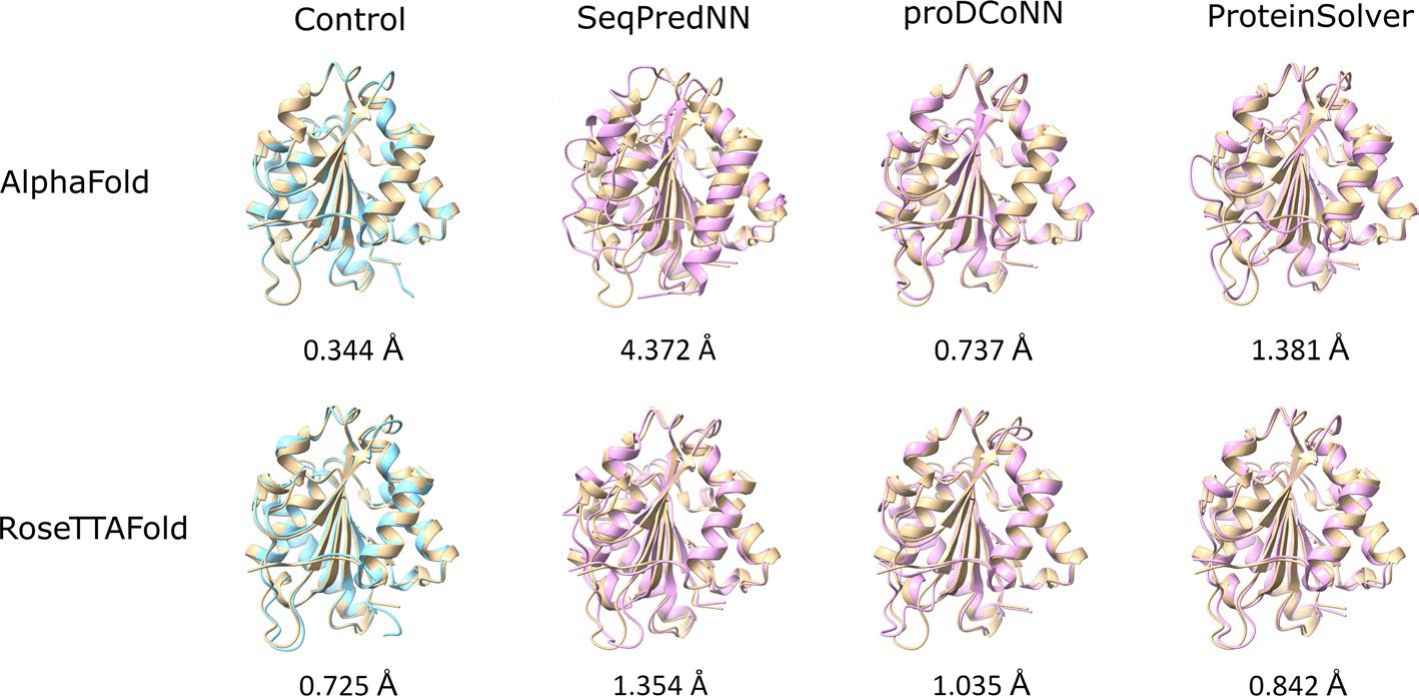
Folded structures of predicted sequences are close to the native structure. The superposition of the control structure predicted from the real sequence (blue) and the crystal structure (tan) is shown, as well as the superposition of the structures predicted for the sequences generated with SeqPredNN, proDCoNN and ProteinSolver (magenta) and the control structure (tan). Structure predictions were performed with AlphaFold (top row) and RoseTTAFold (bottom row). The C_a_–C_a_ RMSD values are shown

We used both AlphaFold and RoseTTAFold structure predictions to show that SeqPredNN produces sequences that largely fold into conformations close to the conformation of the native sequence. Focusing on the small globular *Bacillus* lipase A, a superposition of the predicted and control structures provides a visual impression of the similarity of the predicted structures (Fig. 8).

We compare the folds of sequences generated by SeqPredNN, ProDCoNN and ProteinSolver using predictions by both AlphaFold and RosettaFold. Focusing on the small globular *Bacillus* lipase A, a superposition of the predicted and control structures provides a visual impression of the similarity of the predicted structures (Fig. 8). It is clear that all the models predicted sequences that are expected to fold into the same secondary structures as the native crystal structure, with only small differences in the packing of the alpha-helices and beta-sheets, and in the conformation of loops and tails.

**Fig. 9:**
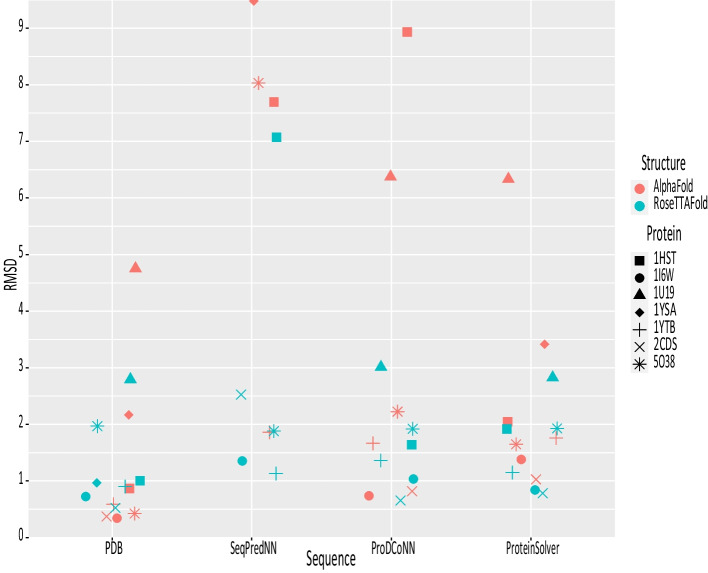
Difference between predicted structures and the native crystal structure. Cα–Cα RMSD of AlphaFold and RoseTTAFold predicted protein structures aligned to the original PDB crystallographic structure. The predicted structures for the native sequences (PDB) serve as a control to compare the SeqPredNN, ProDCoNN and ProteinSolver sequences of the seven proteins. Both the AlphaFold and RoseTTAFold predictions for the ProdCoNN sequence of 1YSA lie outside the plot area with RMSD values of 18.9 and 19.8 Å, respectively. The RoseTTAFold could not predict the structure of the ProteinSolver sequence for 1YSA

Looking at the closeness-of-fit for the seven sequences, it is seen (Fig. 9) that the RMSD values for SeqPredNN largely falls within the same range as the other methods, producing several structures that do not deviate significantly more from the native structure than the error associated with the AlphaFold prediction. It should be noted that the ProteinSolver prediction for GCN4 has a very low sequence complexity, and RoseTTAFold could not predict a structure for this sequence.

It is clear from the compared RMSD values that SeqPredNN produces proteins with structures as close to the native PDB structure as the proteins predicted by ProDCoNN and ProteinSolver and well within the range of error of the structure prediction methods. SeqPredNN, however, has the advantage of generating sequences with a lower identity to the crystallised sequences compared to proDCoNN and ProteinSolver, allowing the design of proteins with identical structures, but more divergent in physicochemical or dynamic properties, a desirable feature in biotechnological applications.

SeqPredNN is easily accessible by download from the GitHub page and can be executed from any workstation with Python 3.9 or higher installed with common, freely available python packages. We include detailed usage instructions for users to generate new sequences, as well as directions to train new models using custom datasets. SeqPredNN can rapidly generate predictions for thousands of structures in two steps: by first executing the *Featurise* and then the *Predict* program from the command line. The source code can be modified freely and adapted to suit different workflows in accordance with the GNU General Public License.

## Conclusion

SeqPredNN is simple, yet powerful neural network implementation for generating protein sequences with desired backbone conformations. Highly accurate structure prediction models confirm that the predicted sequences are likely to fold into the target structure. SeqPredNN produces novel protein sequences that diverge more from natural proteins than sequences generated by the other models, suggesting that it will be more versatile in enzyme design and polymer and material science.

## Availability and requirements

Project name: SeqPredNN

Project home page: https://github.com/falategan/SeqPredNN

Operating system(s): GNU/Linux, Windows

Programming language: Python 3.9

Other requirements: Pytorch, Numpy, SciKit-Learn, Matplotlib, Scipy, Biopython

License: GNU GPL 3

Any restrictions to use by non-academics: N/A.

## Data Availability

The source code, model parameters, and a list of all the protein chains used to train the model are available at https://github.com/falategan/SeqPredNN. All the protein structures used can be downloaded from the Protein Data Bank at https://www.wwpdb.org/ or https://www.rcsb.org/.

## Abbreviations

CI: Confidence interval
PDB: Protein Data Bank
RMSD: Root-mean-square deviation
